# Antihypertensive and Diuretic Effects of the Aqueous Extract of *Colocasia esculenta* Linn. Leaves in Experimental Paradigms

**Published:** 2012

**Authors:** Otari Kishor Vasant, Bhalsing Gaurav Vijay, Shete Rajkumar Virbhadrappa, Nandgude Tanaji Dilip, Mali Vishal Ramahari, Bodhankar Subhash Laxamanrao

**Affiliations:** a *Department of Pharmacology, Rajgad Dyanpeeth’s College of Pharmacy, Bhor, Dist: Pune, India-412 206, Maharashtra, India. *; b *Department of Pharmacology, Poona College of Pharmacy, Bharati Vidyapeeth University, Paud Road, Erandwane, Pune 411 038, Maharashtra, India.*

**Keywords:** Colocasia esculenta Linn, Antihypertensive, Diuretic, Noradrenaline, Flavonoids, Renal artery-occluded hypertension

## Abstract

*Colocasia esculenta *Linn (*CE*) is traditionally used for the treatment of various ailments such as high blood pressure, rheumatic pain, pulmonary congestion, etc. Hence in present study, the effect of aqueous extract of *CE* leaves (AECE) was evaluated for antihypertensive and acute diuretic activity in rats.

Preliminary phytochemical evaluation revealed the presence of carbohydrate, saponins, tannins, and flavonoids in AECE. The animals did not show any sign of toxicity and mortality after the administration of AECE 2000 mg/Kg in acute oral toxicity study. The administration of AECE (100, 200, and 400 mg/Kg/day, p.o.) for six weeks and AECE (10, 20, and 40 mg/Kg, IV) on the day of experiment in renal artery-occluded hypertensive rats and AECE (20 and 40 mg/Kg, IV) in noradrenalin-induced hypertension in rats produced significant (p < 0.05) anti-hypertensive effects. AECE (400 mg/Kg, p.o.) showed positive diuretic activity at 5 h. AECE (200 and 400 mg/Kg, p.o.) significantly increased sodium and chloride content of urine in 5 h and 24 h and additionally potassium in 24 h urine.

Hence, the results of the present study revealed the antihypertensive and weak diuretic activity of AECE. These effects may be attributed due to the ACE inhibitory, vasodilatory, *β*-blocking, and/ or Ca^2+^ channel blocking activities, which were reported for the phytoconstitunts, specifically flavonoids such as vitexin, isovitexin, orientin, and isoorientin present in the leaves of *CE*.

## Introduction

Hypertension is a chronic and asymptomatic to symptomatic disorder characterized by a persistently elevated blood pressure (BP) exceeding 140/90 mmHg or greater (1). Cardiovascular disease alone accounts for nearly 30% of all deaths worldwide and 10% of all years of healthy life lost to disease. In low-income and lower middle-income countries, the corresponding figures are similar to the global ones (27% and 9%, respectively) (2).

Clinically, various antihypertensive drugs have been used to manage hypertension and to alleviate symptoms. However, the efficacy of these drugs is only 40-60% and usually two or more antihypertensive drugs from different categories are needed to be combined to achieve the optimal results and thus, this ultimately increases the cost of treatment and side effects (3). The frequent side effects of synthetic antihypertensive drugs includes dry mouth, dizziness, visual disorders, headache, cough, emotional distress, gastrointestinal disturbance, peripheral circulatory symptoms like cold hands and feet, swollen ankles, *etc.* (4, 5). These distressing side effects can lead to non-compliance and adversely affects health-related quality of life. Therefore, the search for natural, cheaper and non-toxic compound is become necessary (6-8).

Herbal medicines have been commonly used and remain so instead of synthetic drugs because of their possible fewer side effects (9). Most people in the rural areas of the world depend largely on herbs for the treatment of several ailments since the medicinal herbs constitute indispensable components of traditional medicine practice due to the low cost, easy access and ancestral experience (10).

Taro is the common name for edible aroids which are important staple foods in many parts of the world, particularly in Asia and the Pacific Islands. Within the family Araceae, there is one ‘‘true taro’’, namely *Colocasia esculenta *Linn (*CE*) (11). Extract of *CE* leaves has been traditionally used for the treatment of various ailments in Ayurveda and Unani medicine. *CE* is traditionally used in various diseases such as high BP, hepatic disorder, rheumatic pain, pulmonary congestion, ulcer *etc*. The *CE* has been reported for anti-inflammatory (12), hypolipidemic (13), anti-cancer (14), antioxidant (15), and antibacterial (16) activities.

The leaves of *Colocasia eulenta* contains flavonoids such as vitexin, isovitexin, orientin, isoorientin, schaftoside, isoschaftoside (15), luteolin, apigenin (17), vitamins A, B, and C, thiamine riboflavin, niacin, oxalic acid (16), and minerals such as magnesium, calcium, phosphorus, sodium, potassium, iron, zinc, copper, and boron (18, 19). Some of these phytoconstituents are reported for ACE inhibitor (20), hypotensive, anti-inflammatory, and antispasmodic (21), vasodilatory (22), and *β*-blocking, Ca^2+^ channel blocking, and diuretic activity (23).

However, the pharmacological or clinical studies for antihypertensive and diuretic property of *CE* leaves have not yet been reported. Hence, the present research was undertaken to evaluate the antihypertensive and diuretic activity of aqueous extract of *CE* leaves.

## Experimental

*Collection and identification of plant material*

The leaves of *Colocasia esculenta *were collected from Bhor region (Dist. Pune, Maharashtra, India) in December 2009. The plant specimen was authenticated and herbarium was deposited at Botanical Survey of India, Pune, India (BSI/WRC/Tech/2010/592).

*Preparation of aqueous extract of Colocasia esculenta leaves (AECE)*

The leaves were dried under the shade and powdered using a grinder mixer. The powdered material (1000 g) was socked in cold distilled water for 72 h and then filtered. The obtained filtrate was evaporated on water bath to obtain the solid reddish colored dry mass of 26 g (2.60 % w/w). The extract was then preserved in the desiccators (24, 25).

*Drug and Chemicals*

Urethane (Himedia Laboratories Pvt. Ltd., India); Heparin (Merlin Pharma Pvt. Ltd., India); Captopril (Aciten^®^ dispersible tablet 25 mg, Tridoss Laboratories Pvt. Ltd., India); Noradrenaline (Adrenor^®^ injection 2 mg, Samarth Life sciences Pvt. Ltd., India); Hydrochlorothiazide (Aquazide^®^ tablet 12.5 mg, Ajanta Pharmaceuticals Pvt. Ltd., India.); Urea (Research Lab, India); Electrolyte estimation kit (Coral Clinical Systems, India) and all the chemicals of analytical grade were purchased from local vendors from Pune, India.

*Preparation of drug solution*

AECE, Aciten^®^ tablet, and Aquazide^®^ tablet were powder triturated and suspended in 0.5% CMC in distilled water. Adrenor^®^ injection was diluted with distilled water. Urea was powder triturated and dissolved in distilled water. All solutions were prepared freshly and stored in glass bottles.

*Animals*

Adult Swiss albino female mice (20-30 g) used for acute oral toxicity (AOT) study and adult male Wistar rats (200-250 g) (Grade II) used for the other studies were procured from National Toxicology Center, Pune, India. Animals were housed in groups of 5-6 in standard polypropylene cages with wire mesh top at standard environmental condition at temperature of 25 ± 2ºC and relative humidity of 45-55% under 12 h: 12 h light:dark cycle in the institutional animal house. Animals had free access to standard pellet rodent diet (Lipton India Ltd.; Mumbai, India) and water was provided *ad libitum*. All experiments were carried out between 08:00 to 16:00. The experimental protocol was approved by the Institutional Animal Ethics Committee of Rajgad Dnyanpeeth’s College of Pharmacy, Bhor, Dist. Pune, India (RDCOP/IAEC/10/03).

*Preliminary phytochemical evaluation of AECE*

AECE was subjected for the qualitative analysis by using the standard phytochemical tests to evaluate the presence of various phytoconstituents (24).

**Figure 1:**
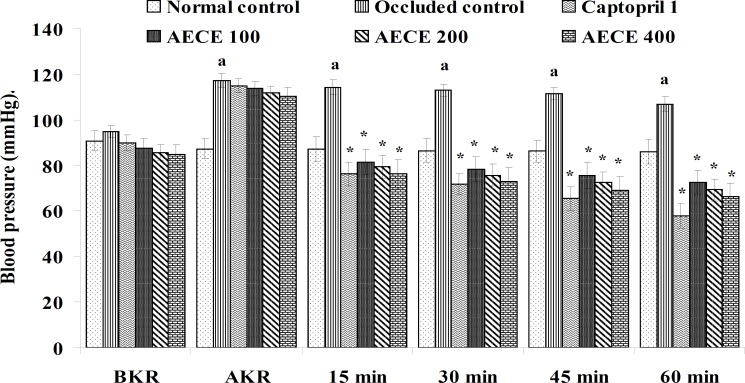
Effect of AECE on BP in renal artery-occluded hypertensive rats. Values are expressed as mean ± SEM (n = 6). ^a^p < 0.05 as compared with normal control (Student t-test), ^*^p < 0.05 as compared with occluded control (one-way ANOVA followed by Dunnett’s test).

*Acute oral toxicity study (AOT)*

Healthy adult Swiss female mice (20-30 g) were subjected to AOT studies as per Organization for Economic Co-operation and Development (OECD) guidelines 2001 (AOT-423). Animals were observed individually after dosing at least once during the first 30 min, periodically during the first 24 h, with special attention given during the first 4 h, and daily thereafter, for a total of 14 days. The changes in skin, fur, eyes, mucous membranes, respiratory, circulatory, autonomic, central nervous system, somatomotor activity and behaviour pattern were noted (26).

**Figure 2:**
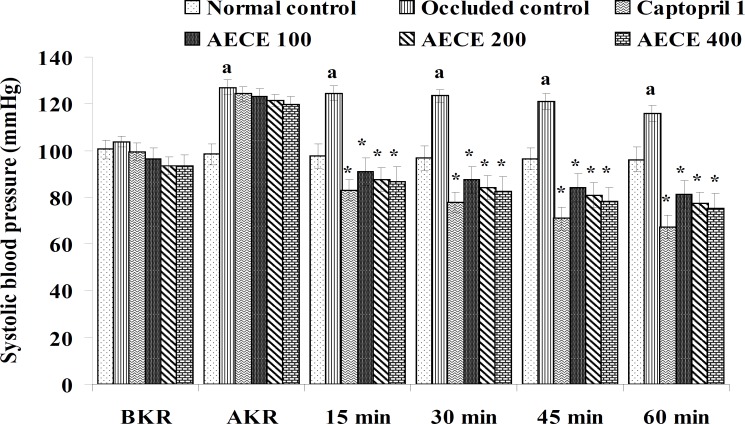
Effect of AECE on SBP in renal artery-occluded hypertensive rats. Values are expressed as mean ± SEM (n = 6). ^a^p < 0.05 as compared with normal control (Student t-test), ^*^p < 0.05 as compared with occluded control (one-way ANOVA followed by Dunnett’s test).

*Effect of AECE in renal artery-occluded hypertensive rats*

*Experimental design*

Male Wistar rats (200-250 g) were randomly divided into six groups (n = 6) and treated as follows:

Normal control group: distilled water (10 mL/Kg, p.o.); occluded control group: distilled water (10 mL/Kg/p.o.); captopril 1: captopril (1 mg/Kg, IV) on day of experiment; AECE 100, 200, and 400 groups: AECE (100, 200, and 400 mg/Kg/day, p.o.) for 6 weeks and AECE (10, 20, and 40 mg/Kg, IV) on the day of experiment respectively.

**Figure 3:**
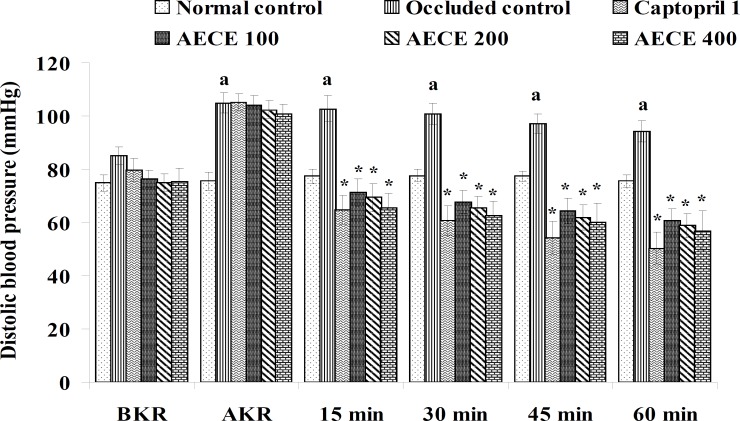
Effect of AECE on DBP in renal artery-occluded hypertensive rats. Values are expressed as mean ± SEM (n = 6). ^a^p < 0.05 as compared with normal control (Student t-test), ^*^p < 0.05 as compared with occluded control (one-way ANOVA followed by Dunnett’s test).

Twenty-four h after the last treatment, animals were anaesthetized by urethane (1.25 g/Kg, IP). A small incision was given on the left side of the peritoneal cavity of animal to expose the left kidney. The renal artery was occluded for 4 h by using renal bulldog clamp. The jugular vein was cannulated for the drug treatments. The carotid artery was cannulated and connected to the POWER LAB (8/30, AD Instruments, Australia) through the pressure transducer. After the stabilization of BP, the renal bulldog clamp was removed, BP was stabilised for 10 min, and immediately respective treatments were given through the jugular vein.

The parameters like BP, systolic BP (SBP), diastolic BP (DBP) and mean arterial BP (MABP) were recorded for each animal after (AKR) and before (BKR) the removal of renal bulldog clamp, and at 15, 30, 45, and 60 min after the IV treatments. All the parameters for normal control group were recorded without clamping the renal artery (25, 27, 28).

*Effect of AECE on noradrenaline-induced hypertension in rats*

*Experimental design*

Male Wistar rats (200-250 g) were randomly divided into five groups (n = 6) and treated as follows:

Normal control group: distilled water (1 mL/Kg, IV); NA (noradrenaline) group: distilled water (1 mL/Kg, IV); AECE 10, 20, and 40 groups: AECE (10, 20, and 40 mg/Kg, IV) on the day of experiment respectively. Animals were anaesthetized using urethane (1.25 g/Kg, IP). The jugular vein was cannulated for the drug treatments. The carotid artery was cannulated and connected to the POWER LAB (8/30, AD Instruments, Australia) through the pressure transducer. To the animals from all groups, except normal control group, noradrenaline (1 μg/Kg, IV) was administered through jugular vein for the induction of hypertension. Then, after the stabilisation of BP for 10 min, the respective treatments were given. The parameters like BP, SBP, DBP, and MABP were recorded for each animal after stabilisation of BP (STB) and at 15, 30, 45, and 60 min after the IV treatments (29, 30).

**Figure 4:**
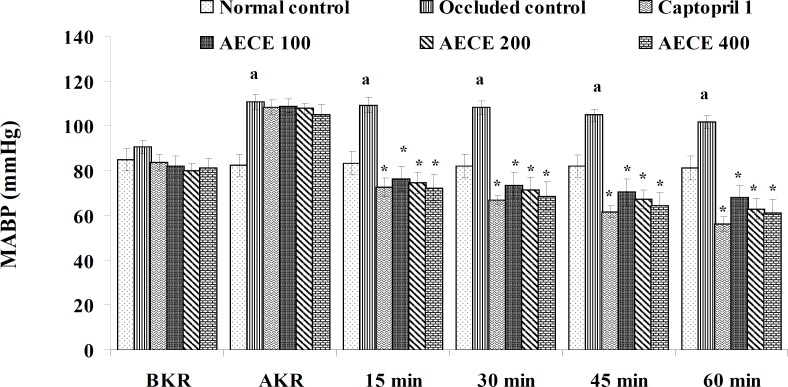
Effect of AECE on MABP in renal artery-occluded hypertensive rats. Values are expressed as mean ± SEM (n = 6). ^a^p < 0.05 as compared with normal control (Student t-test), ^*^p < 0.05 as compared with occluded control (one-way ANOVA followed by Dunnet’s test).

*Acute diuretic activity of AECE in rats*

*Experimental design*

Male Wistar rats (200-250 g) were randomly divided into six groups (n = 6) and treated as follows:

Fifteen h prior to the experiment, food and water was withdrawn. Normal control group: distilled water (10 mL/Kg, p.o.); Urea group: urea (1 g/Kg, p.o.); HTZ group: hydrochlorothiazide (1 mg/Kg, p.o.); AECE 100, 200, and 400 groups: AECE (100, 200, and 400 mg/Kg, p.o.) respectively. Additionally, normal saline (5 mL/100 g, p.o.) was given by gavage to the animals from all treatment groups. Three animals per group were placed in metabolic cages provided with a wire mesh bottom and a funnel to collect the urine.

*Estimations of urine volume, sodium, potassium, chloride content of urine and Lipschitz values*

Urine volume excreted per 100 g body weight was calculated for each group and Lipschitz value, the ratio of T/U, where T was the response of the treatment group and U that of urea treatment was calculated. Indices of 1.0 and more were regarded as a positive effect. With potent diuretics, Lipschitz-value of 2.0 and more can be found. Calculating this index for the 24 h excretion period as well as for 5 h indicates the duration of the diuretic effect. Similar to the urine volume, quotients were calculated for sodium excretion. Saluretic drugs, like hydrochlorothiazide, show Lipschitz-value around 1.8, whereas loop diuretics (or high ceiling diuretics) like furosemide reach value of 4.0 or more. The sodium, potassium, and chloride content of urine were analyzed at 5 h and 24 h after the treatment by using respective standard electrolyte estimation kit procedures in auto-analyzer (28, 31, 32).

**Figure 5:**
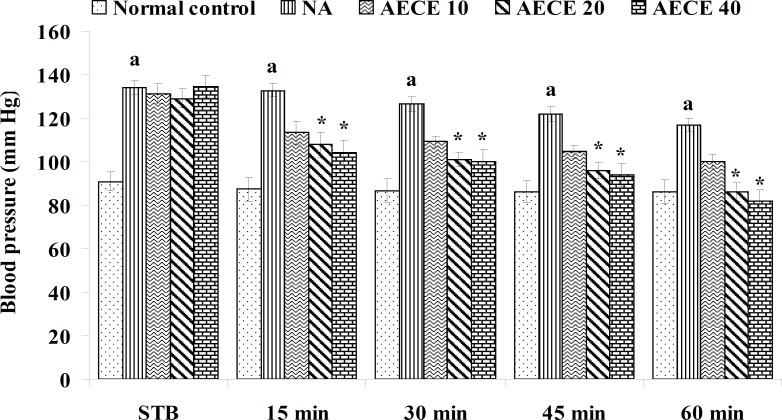
Effect of AECE on BP in noradrenaline-induced hypertension in rats. Values are expressed as mean ± SEM (n = 6). ^a^p < 0.05 as compared with normal control (Student t-test), ^*^p < 0.05 as compared with NA group (one-way ANOVA followed by Dunnet’s test).

*Statistical analysis*

The values were expressed as mean ± SEM (n = 6). The statistical significance was assessed using student t-test or one-way analysis of variance (ANOVA) followed by Dunnett’s test and p < 0.05, p < 0.01, and p < 0.001 were considered to be statistically significant.

## Results and Discussion

*Preliminary phytochemical evaluation of AECE*

Preliminary phytochemical evaluation revealed the presence of carbohydrate, saponins, tannins, and flavonoids in AECE.

*Acute oral toxicity study (AOT)*

The animals treated with AECE did not show any signs of toxicity and mortality during the first 24 h and up to 14 days after the administration of a limit dose of 2000 mg/Kg. Furthermore, there was not any change in the skin, fur, eyes, mucous membranes, respiratory, circulatory, autonomic, central nervous system, somatomotor activity and behavior pattern of the animals. Hence, for the further pharmacological studies doses selected were 100, 200, and 400 mg/Kg for oral and 10, 20, and 40 mg/Kg for intravenous administration.

**Figure 6:**
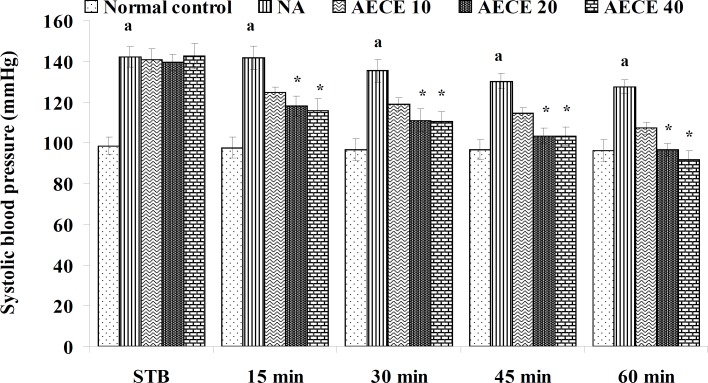
Effect of AECE on SBP in noradrenaline-induced hypertension in rats. Values are expressed as mean ± SEM (n = 6). ^a^p < 0.05 as compared with normal control (Student t-test), ^*^p < 0.05 as compared with NA group (one-way ANOVA followed by Dunnett’s test).

*Effect of AECE in renal artery-occluded hypertensive rats*

Removal of bulldog clamp showed a significant (p < 0.05) increase in BP, SBP, DBP, and MABP in occluded control group as compared with normal control group. Treatment with AECE (100, 200, and 400 mg/Kg/day, p.o.) for six weeks and (10, 20, and 40 mg/Kg, IV) on the day of experiment showed significant (p < 0.05) and dose dependent decrease in BP, SBP, DBP, and MABP at different time intervals when compared with occluded control group. Captopril (1 mg/Kg, IV) showed significant (p < 0.05) reduction in BP, SBP, DBP, and MABP as compared with occluded control (Figures 1-4).

**Figure 7:**
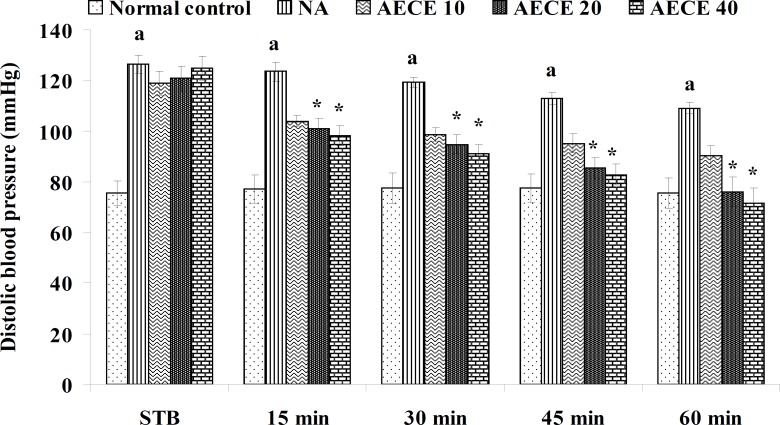
Effect of AECE on DBP in noradrenaline-induced hypertension in rats. Values are expressed as mean ± SEM (n = 6). ^a^p < 0.05 as compared with normal control (Student t-test), ^*^p < 0.05 as compared with NA group (one-way ANOVA followed by Dunnett’s test).

*Effect of AECE on noradrenaline-induced hypertension in rats*

**Figure 8:**
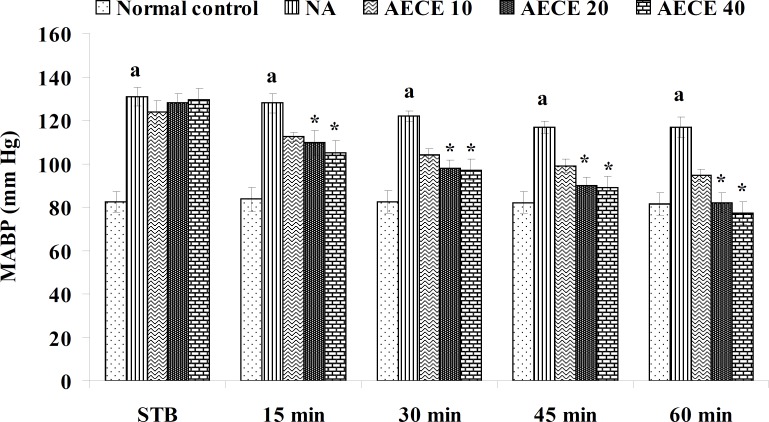
Effect of AECE on MABP in noradrenaline-induced hypertension in rats. Values are expressed as mean ± SEM (n = 6). ^a^p < 0.05 as compared with normal control (Student t-test), ^*^p < 0.05 as compared with NA group (one-way ANOVA followed by Dunnett’s test).

The administration of noradrenaline (NA) showed significant (p < 0.05) increase in BP, SBP, DBP, and MABP as compared with normal control group. The treatment with AECE (20 and 40 mg/Kg, IV) showed significant (p < 0.05) and dose dependent decrease in BP, SBP, DBP, and MABP at 15 to 60 min as compared with NA group. AECE (10 mg/Kg, IV) showed insignificant effects in this regard (Figure 5-8).

*Acute diuretic activity of AECE in rats*

*Urine volume*

The administration of urea (1 g/Kg, p.o.) and hydrochlorothiazide (10 mg/Kg, p.o.) showed a significant (p < 0.01 and p < 0.001 respectively) increase in urine volume as compared with normal control group at 5 and 24 h.

The administration of AECE (200 and 400 mg/Kg) showed significant (p < 0.05 and p < 0.01 respectively) increase in urine volume as compared with normal control group at 5 h. AECE (100 mg/Kg) showed insignificant effects in this regard.

**Figure 9:**
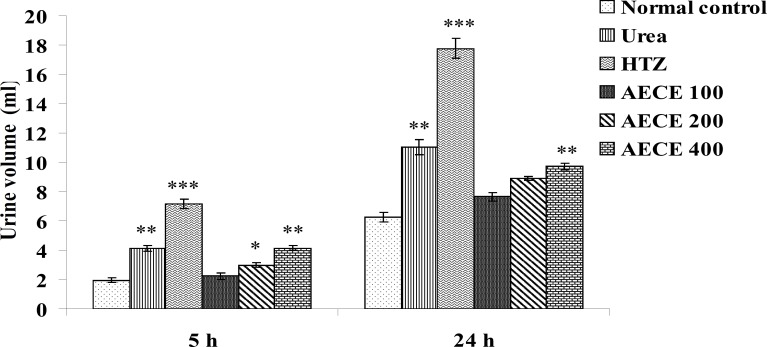
Effect of AECE on urine volume. Values are expressed as mean ± SEM (n = 6). ^*^p < 0.05, ^**^p < 0.01, ^***^p < 0.001 as compared with normal control (one-way ANOVA followed by Dunnett’s test).

The administration of AECE (400 mg/Kg) showed significant (p < 0.05) increase in urine volume when compared with normal control group at 24 h. AECE (100 and 200 mg/Kg) showed insignificant effects in this regard (Figure 9).

*Sodium content*

The administration of urea (1 g/Kg, p.o.) and hydrochlorothiazide (10 mg/Kg, p.o.) showed significant (p < 0.001) increase in sodium content of urine as compared with the normal control group at 5 h. The administration of AECE (200 and 400 mg/Kg, p.o.) showed significant (p < 0.05 and p < 0.01 respectively) increase in sodium content of urine as compared with normal control group at 5 h.

**Figure 10:**
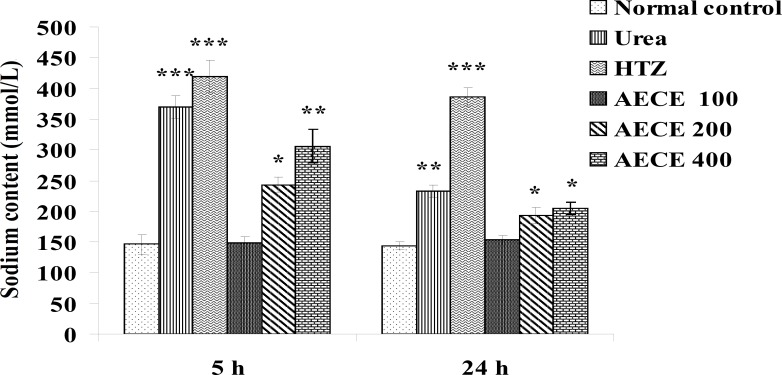
Effect of AECE on sodium content of urine. Values are expressed as mean ± SEM (n = 6). ^*^p < 0.05, ^**^p < 0.01, ^***^p < 0.001 as compared with normal control (one-way ANOVA followed by Dunnett’s test).

The administration of urea (1 g/Kg, p.o.) and hydrochlorothiazide (10 mg/Kg, p.o.) showed significant (p < 0.01 and p < 0.001 respectively) increase in sodium content of urine as compared with normal control group at 24 h. The administration of AECE (200 and 400 mg/Kg, p.o.) showed significant (p < 0.05) increase in sodium content of urine as compared with normal control group at 24 h. AECE 100 mg/Kg showed insignificant effects in this regard at 5 h and 24 h (Figure 10).

*Chloride content*

The administration of urea (1 g/Kg, p.o.) and hydrochlorothiazide (10 mg/Kg, p.o.) showed significant (p < 0.01 for both) increase in chloride content of urine as compared with the normal control group at 5 h. The administration of AECE (200 and 400 mg/Kg, p.o.) showed significant (p < 0.05 and p < 0.01 respectively) increase in chloride content of urine as compared with the normal control at 5 h.

**Figure 11:**
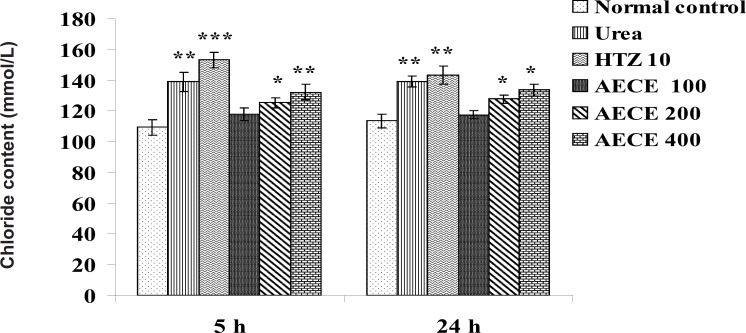
Effect of AECE on chloride content of urine. Values are expressed as mean ± SEM (n = 6). ^*^p < 0.05, ^**^p < 0.01, ^***^p < 0.001 as compared with the normal control (one-way ANOVA followed by Dunnett’s test).

The administration of urea (1 g/Kg, p.o.) and hydrochlorothiazide (10 mg/Kg, p.o.) showed significant (p < 0.01) increase in chloride content of urine as compared with the normal control group. AECE (200 and 400 mg/Kg, p.o.) showed significant (p < 0.05) increase in chloride content of urine as compared with normal control group at 24 h. AECE (100 mg/Kg) showed insignificant effects in this regard at 5 and 24 h (Figure 11).

*Potassium content*

**Figure 12:**
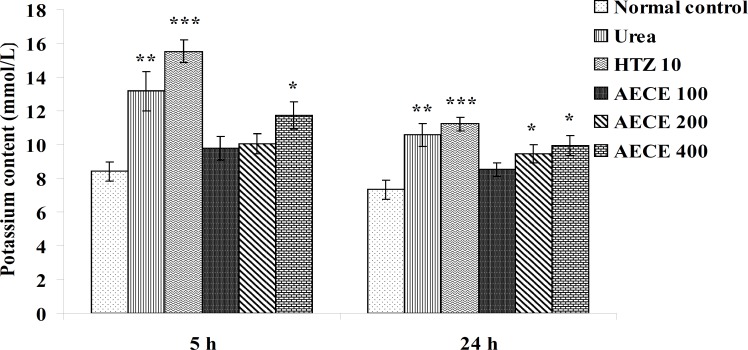
Effect of AECE on potassium content of urine. Values are expressed as mean ± SEM (n = 6). ^*^p < 0.05, ^**^p < 0.01, ^***^p < 0.001 as compared with the normal control (one-way ANOVA followed by Dunnett’s test).

The administration of urea (1 g/Kg, p.o.) and hydrochlorothiazide (10 mg/Kg, p.o.) showed significant (p < 0.01 and p < 0.001 respectively) increase in potassium content of urine as compared with the normal control group at 5 h and 24 h. The administration of AECE (400 mg/Kg, p.o.) showed significant (p < 0.05) increase in potassium content of urine as compared with the normal control at 5 h and 24 h. The administration of AECE (200 and 400 mg/Kg, p.o.) showed significant (p < 0.05) increase in potassium content of urine as compared with the normal control group at 24 h. AECE (100 mg/Kg) showed insignificant effects in this regard at 5 h and 24 h (Figure 12).

*Lipschitz values*

administration of AECE (400 mg/Kg, p.o.), but not (100 and 200 mg/Kg, p.o.) showed a positive diuretic effect at 5 h, as evident from the Lipschitz value for the urine volume. AECE, at all tested doses, did not show positive diuretic effect at 24 h. Furthermore, the administration of AECE, at all tested doses, did not found to be saluretic, loop diuretic, or high ceiling diuretic, as evident from the Lipschitz value for sodium excretion (Table 1).

## Discussion

Two kidney one clamp (2K1C) model, which mimics the renal artery constriction as in humans, was used in the present study (33). Acute renal hypertension in rats can be induced through clamping the renal artery for 4 h that leads to the kidney ischemia and causes elevation of BP by the activation of the rennin-angiotensin system. After the reopening of vessels, the accumulated rennin is released into the circulation (25). Renin acts on the angiotensinogen to form angiotensin I which is then converted to angiotensin II by angiotensin-converting enzyme (ACE). ACE also catalyzes inactivation of the vasodilator bradykinin (33, 34).

**Table 1:** Lipschitz values

**Group**	**Lipschitz values (T/U)**			
	**For urine volume**		**For sodium excretion**	
	**5 h**	**24 h**	**5 h**	**24 h**
**Normal control**	-	-	**-**	**-**
**Urea**	-	-	**-**	**-**
**HTZ**	1.72	1.61	1.53	1.64
**AECE 100**	0.54	0.69	0.40	0.65
**AECE 200**	0.72	0.80	0.68	0.82
**AECE 400**	1.00	0.88	0.82	0.87

Angiotensin II causes the activation of AT_1_ and AT_2_ receptors, stimulation of adrenal cortex to secrete aldosterone, and inhibition of prostaglandin synthesis which is secondary but also relevant (35). It causes constriction of arterioles so that the peripheral resistance increases and thus the BP. In addition, angiotensin II causes the constriction of afferent arterioles in the kidney, so that the glomerular filtration reduces this result in retention of water and salts. Additionally, aldosterone increases the reabsorption of sodium from the renal tubules that increases the volume of ECF, which in-turn increases the BP (1). It has been suggested that most of the changes in 2K1C model affecting the hemodynamic and structural alterations are caused through the increased ACE activity and generation of angiotensin II (33). ACE inhibitors have a potent antihypertensive effect in both human and animal (36). Captopril, used as a reference standard, inhibits the ACE and also causes the depression of baroreceptor sensitivity which reduces the activity of vasomotor center, leading to the reduction of vasopressor effect (27).

Despite understanding that BP remains a strong modifiable risk factor for the cardiovascular (CVS) morbidity, the increasing incidence of stroke, renal disease, and heart failure (HF) suggests that the control of BP has been inadequate. The isolated systolic hypertension (ISH), *i.e*. SBP >140 mmHg with DBP < 90 mmHg (37) and prehypertension, particularly high normal BP (SBP of 130 to 139 or DBP of 85 to 89 mmHg) is associated with an increased risk of cardiovascular disease (CVD) mortality. Prehypertension SBP is a most relevant parameter in coronary heart disease (CHD). It appears that DBP and MABP may be slightly more informative in predicting stroke mortality and SBP and MABP, in predicting CHD mortality. Hypertension and higher levels of individual BP parameters including SBP, DBP, and MABP were positively associated with all-cause, stroke, and CHD mortality (38). Hence in the present study, BP, SBP, DBP and MABP were evaluated.

In the present study, the occlusion of renal artery induced hypertension in rats, as it is evident from the significant increase in BP, SBP, DBP, and MABP, in occluded control group as compared to the normal control group. The renal artery occlusion-induced hypertension in rats was significantly and dose-dependently decreased after the treatment with AECE at different time intervals as compared with the occluded control group. Captopril produced significant reduction in BP, SBP, DBP, and MABP as compared to the occluded control group.

Noradrenaline (NA), a sympathomimetic drug, has strong agonistic activity on *α*_1_, *α*_2_, *β*_1 _adrenergic receptors but it lacks activity on *β*_2-_receptor. NA shows hypertensive action through increasing the vascular tone, total peripheral resistance, and coronary blood flow. NA causes vasoconstriction as a result of its *α*-agonistic activity. It consequently increases the peripheral vascular resistance in most vascular beds and decreases the renal blood flow causing a raise in both SBP and DBP. The cardiac output is either unchanged or slightly affected. NA-induced reflex cardioinhibition leads to an increase in the reflex vagal activity that decreases the rate and force of the heart beat, which in-turn antagonizes the weak *β* stimulating effect of NA in heart (39, 40). NA has little or no action on the basic ventricular pacemaker. As compared with epinephrine and isoprenaline, NA shows little or no effect on cardiac rhythmicity. The infusion of NA increases systolic, diastolic, and usually pulse pressure (39, 41-43).

In the present study, the administration of NA (1 µg/Kg) showed significant increase in BP, SBP, DBP, and MABP as compared to the normal control group, which was significantly decreased after the treatment with AECE at doses of 200 and 400 mg/Kg. Hence, the results of present study revealed that the antihypertensive effect of AECE may be due to the vasodilation.

Herbal plants used as diuretics in traditional medicinal system might be useful in the treatment of hypertension (9). One of the primary functions of kidneys is to regulate Na^+^ and water excretions, and consequently, they play a dominant role in the long-term control of BP (39, 44). Diuretics act within the kidney and promote the loss of fluid from the body. To be clinically effective, however, such compounds must induce the loss of sodium. This helps to reduce the volume of blood circulating through the cardiovascular system (45).

In the present study, AECE at a dose of 400 mg/Kg showed positive diuretic activity at only 5 h, as evident from the Lipschitz values for urine volume. Furthermore, AECE at 200 and 400 mg/Kg showed significant increase in sodium and chloride content of urine at 5 h and sodium, potassium and chloride content of urine at 24 h. From the Lipschitz value for the sodium excretion AECE, at all tested doses, did not prove to be saluretic, loop diuretic, or high ceiling diuretic. Hence, the results revealed the weak diuretic activity of AECE with a shorter duration of action at a higher tested dose.

The preliminary phytochemical investigations in the present study revealed the presence of flavonoids, saponins, alkaloids, and tannins in AECE. The presence of flavonoids such as vitexin, isovitexin, orientin, isoorientin, schaftoside, isoschaftoside (15) luteolin and apigenin (17) in the plant has been previously reported. The flavonoids vitexin and isovitexin showed *in-vitro* inhibition of ACE activity (20). The *in-vitro* and *in-vivo* hypotensive effect of vitexin was reported earlier (21). The isoorientin showed myolytic activity on uterine smooth muscle that may be due to the inhibition of phosphodiesterase which consequently increases the cellular concentration of cyclic nucleotides which may causes vasodilation (22). Orientin, isoorientin, vitexin inhibits ACE (46). Vitexin and vitexin rhamnoside may also have *β*-blocking, Ca^2+^ channel blocking, and diuretic effects (23).

The results of the present study were suggested that the flavonoids present in AECE may be responsible for the antihypertensive and weak diuretic effects. The observed antihypertensive effect was possibly due to the inhibition of ACE, functioning as an antagonist at vascular α-receptors, inhibition of phosphodiesterase or the direct action on vascular endothelium to increase the release of EDRF, and thereby, producing the vasodilation. AECE may by inhibiting ACE produce diuretic effect which can be further contributed to its antihypertensive effect. Further studies are necessary to be performed for the purification, isolation and characterization of the phytoconstituents responsible for the antihypertensive and diuretic effect and to explore the exact mechanism of the action.
